# Toddler–teacher interaction and teachers’ sensitivity as predictors of toddler’s development during COVID-19: Stability or change over time

**DOI:** 10.3389/fpsyg.2023.1161947

**Published:** 2023-04-17

**Authors:** SoJung Seo, JiYeon Song

**Affiliations:** Department of Child & Family Studies, Kyung Hee University, Seoul, Korea

**Keywords:** toddler–teacher interaction, toddlers’ development, COVID-19, teacher’s sensitivity, early childhood education

## Abstract

This study examined the stability and change patterns among toddlers’ interactions with their teachers, teachers’ sensitivity, and toddlers’ development during the COVID-19 pandemic and the three plausible paths were tested to identify which of the study variables affected the development of toddlers in subsequent periods over time. The subjects of this study were 63 toddlers and 6 head teachers who attended a subsidized child care center, located in Kyunggi province, Korea. In order to carry out the research objectives, a non-experimental survey research design was undertaken, and the qualitative data was obtained via on-site observations by trained researchers. With regard to continuity and change patterns among the study variables toddlers who had been actively involved in initiating their verbal interactions with teachers showed more verbal interactions with their teachers even after 4 months passed. Also, it was found that the early (T1) social disposition of toddlers and the behavioral interaction that toddlers had initiated with teachers revealed a significant effect, supporting each of the three models, which are simultaneous, cumulative, and complex paths. The main results of this research support the contention that the interaction patterns vary by contexts of subject, time, and history, indicating that it would be useful to understand new competencies required for teachers within the context of the multi-faceted ramifications of the pandemic on toddler development.

## Introduction

As of November 2022, the number of COVID-19 infections globally exceeded 6.1 billion (World Health Organization, 2022) and Korea is no exception, showing that over 24 million cases have been reported in South Korea to date (Korea Disease Control and Prevention Agency, 2022). As this worldwide crisis turned into a prolonged pandemic, people responded properly with various social distancing guidelines, such as wearing a mask and following recommendations for home-based work over the past 3 years.

However, it has been expected that the extended crisis of COVID-19 would have several direct or indirect impacts on a multi-faceted society in general, and specifically in the arena of Early Childhood Education and Care (ECEC). The concern centers on the potential challenges and limitations that young children face and deal with in their daily routine, implying that young children who are in the blind spot of care are the most vulnerable to psychological and behavioral development than any other life cycle due to the unprecedented changes associated with COVID-19 (Bhutta et al., 2017; Benner and Mistry, 2020; United Nations, 2020; Green et al., 2021).

In line with the challenges and changes associated with ECEC and addressed herein, most ECEC facilities were closed temporarily and emergency care was only implemented for young children and their families in need based upon social distancing guidelines mandated from the Korean government when the COVID-19 situation worsened. As a result, the rate of enrollment in ECEC facilities after the outbreak of COVID-19 dramatically declined from 88.5 to 44.5%, as compared to that of previous year of 2020 in Korea (Korean Ministry of Health and Welfare, 2021). Also, outdoor and large group activities were limited and the opportunities to interact with teachers or peers decreased during this pandemic period of COVID-19. Thus, the contention that these immediate and robust changes that young children faced in their daily routine may have hindered them in developing social relationships with significant others (teachers and peers) has become highlighted and convincing enough to trigger not only the need for a new ECEC system but also a consideration of the appropriate role of teachers in the post-COVID-19 period (NAEYC, 2020).

### The impact of COVID-19 on toddlers from a life-span perspective

To date, there has been a near void in the related research on the direct or indirect impacts of COVID-19 on young children, though some empirical studies have been burgeoning to explore potential links between the robust changes related to the social distancing measures of COVID-19 and early development among young children in ECEC settings. One of the research endeavors that need to be addressed is rooted in the notion of the life-span perspective which is a theoretical approach to grasp the multiple influences of the daily ecological context as well as the larger societal and broader socio-historical context. Delineated from Elder’s life course theory (Elder, 1998), it focuses on the simultaneous or potential multi-faceted impact of socio-historical events (e.g., COVID-19) through the lives of individuals depending on their current developmental stage. From this point of view, it is advisable to employ this life-span perspective to fully understand the impact of ECEC settings on young children in their daily routine during COVID-19 as a significant socio-historical event.

In line with the life-span perspective, the scant existing research has shown contradictory findings about the impact of COVID-19 on young children. On the one hand, the restrictive social distancing guidelines with no outdoor activities exacerbated the use of digital media by young children by allowing them to spend much more time with it (Jiao et al., 2020). In support of Jiao et al.’s (2020) findings, Gupta and Jawanda (2020) also found that decreased physical activity has negatively affected various developmental areas, such as lowered immunity, reduced change to experience socialization, and increased aggressive behaviors in young children. Furthermore, it is important to address the research evidence that toddlers wearing a mask initiated interactions less frequently with peers than when not wearing a mask (Green et al., 2021). From neurobehavioral and social aspects, they were more likely to interrupt psychological stability (United Nations, 2020).

On the other hand, a few studies revealed the positive effects of COVID-19 on young children who have been cared for by non-maternal caregivers or teachers in ECEC settings during the pandemic period. Bredeveien (2020) found that dense individual interactions with a young child-teacher ratio per class, which dramatically decreased due to the drop in the ECEC facility enrollment rate during the pandemic, improved the quality of center-based childcare settings. As Handal (2020) asserted, the immediate and robust changes due to the COVID-19 resulted in a turning point of the structural characteristics of the ECEC field, enhancing the ECEC quality to some extent.

In the same vein, recent research by Seo and Song (2021) found similar patterns in young children’s initiation to interact with their teachers as an active social agent, as compared with those observed before the COVID-19 outbreak (Song and Seo, 2021). From a developmental perspective, there were no negative effects of COVID-19 on the levels of developmental outcomes of gross and fine motor development as well communication ability of infants (aged 12 months old), as compared with those test results before the COVID-19 pandemic (Imboden et al., 2021). This finding, in particular, needs to be examined more deeply for possible diverse paths that may mitigate or accelerate the negative impacts of the pandemic along the early developmental trajectory of young children (Benner and Mistry, 2020).

### Toddlers’ outcomes as related to historical events in developmental trajectories

As Bhutta et al. (2017) asserted, young children are most vulnerable to multiple stressors (e.g., COVID-19), and young children’s development could have been substantially affected within the nested contexts of the pandemic. The claim by Bhutta et al. (2017) is also supported by the findings from a previous study by Sprang and Silman (2013) who addressed the plausible linked mechanism between internal and external factors surrounding young children. Specifically, behavior problems in children increased shortly after the outbreak of the global SARS epidemic, and academic achievement had noticeably declined after the Great Recession (Sprang and Silman, 2013). The effects of certain historical events, such as SARS, 9/11, or COVID-19, may be considered as a critical turning point in the developmental trajectory of a child. Furthermore, the effects of teacher–infant interactions within the ECEC contexts needs to be specifically investigated as a positive factor that buffers the developmental risk of young children with low socio-economic status (SES) characteristics (Prime et al., 2020; Imboden et al., 2021).

Coupled with the effects of historical events on child development over time, in recent decades research findings have generally agreed that early childhood is a critical time for providing a variety of developmental appropriate stimuli through continuous, quality interactions with primary caregivers (Seo et al., 2018). It is widely accepted that ECEC quality cannot exceed the quality of the teachers who provide both the education and care for young children (Seo et al., 2016). In the midst of this discussion, a great deal of attention and support from the research arena have centered on the role of teachers’ sensitivity within the context of their interactions with young children in ECEC settings.

One noteworthy finding is the longitudinal study by the Eunice Kennedy Shriver National Institute of Child Health and Human Development (NICHD) which found that infants’ interactions with their teachers at the age of 24 months predicted social development at the age of 3 years (NICHD Early Child Care Research Network, 1996). Also, cognitive and language development improved later in life if toddlers experienced sensitive interactions with their teachers (Klein and Feldman, 2007), and language development was noticeably improved when the teacher provided the verbal model and emotional support tailored to toddlers’ developmental needs (Cadima et al., 2010).

### Teachers’ interaction and sensitivity in ECEC during the pandemic

It is striking that the claim from the most recent study by Davies et al. (2021) is that teachers’ sensitivity to their interactions with young children played a significant role in language development and executive function among young children (aged 8–36 months old) in ECEC settings. On the continuum of findings obtained from western studies, several Korean studies have found that teachers with higher levels of sensitivity tended to interact with their young children in more responsive, reflective, and related ways and that, in turn, positively affected young children’s cognitive and language development compared to their counterparts who had interactions with teachers with lower levels of sensitivity.

However, a few studies cited earlier took an approach of focusing on the concurrent effects of teachers’ interactions with their young children, and research endeavors need to explore various pathways about how teachers’ interactions with young children might predict early or later development in young children. It is necessary to examine the effects of a time lapse on young children’s developmental outcomes.

This study was guided by the pioneering research of Bornstein and Tamis-Lemonda (1990) who proposed three potential models of young children’s development through the relationship between the mother and infant. The first model suggests the lasting ripple effect from the way in which the caregiver initially interacts with the infant, even after a time lapse. The second model assumes the concurrent view that the caregiver interaction has an immediate effect on the infant. The last model highlights both immediate and long-term effects of mothers’ interactions with infants because mothers’ early and later interaction patterns work in more complex and intertwined ways, affecting how infants interact with their mothers as well. Thus, taken into account the possible three models of Bornstein and Tamis-Lemonda (1990), it would be meaningful to lend empirical support to explore overarching research questions of interest in this study about early development among young children who experienced the socio-historical event of the pandemic COVID-19. Furthermore, multi-faceted circumstances associated with COVID-19 would affect the teacher-child interaction in ECEC settings.

To discuss interaction patterns between teachers and young children, this important issue will be centered on the premise that it is necessary to understand the traits of interaction within the context of the teacher-child relationship in nature. Grounded in Sameroff’s transactional theory (Sameroff and Mackenzie, 2003), researchers have explored the plausible links between traits of child (e.g., child’s age, temperament) and those of social contexts (e.g., caregivers, non-maternal infant care) and found an explanation of how each of the traits interplay with developmental outcomes in young children.

This notion by Sameroff’s transactional theory is in line with the compelling research evidence that has been accumulated over the past decades to take into account the concepts of stability and changes over time. The concept of stability in caregiving refers to the consistency of an individual’s behavior over time. Furthermore, continuity or change means the consistency of the whole group’s behavioral pattern (Bornstein and Tamis-LeMonda, 1990). Intuitively, during the period of the pandemic crisis, the interaction patterns might change or persist as a function of traits related to covariates of teacher-infant interactions. Thus, this proposition needs to be empirically supported or tested from a longitudinal perspective within the Asian context of ECEC.

To date, the very few western studies that have been conducted with significant evidence have found the frequency of interaction attempts of toddlers with their teacher was 18% of the observation duration at 1 year, but it was only 6% at the age of three, showing a change in the interaction pattern with the teacher decreasing as the child’s age increased [NICHD Early Child Care Research Network, 1998; HHS (US Department of Health and Human Services, National Institute of Child Health and Human Development), 2006]. For teachers of Korean studies, the frequency of their interactions with toddlers decreased over time, while the types of teachers’ interactions with infants maintained stability during the observation period (Ha and Seo, 2011; Seo and Song, 2021).

Taken as a whole, a life-course perspective and transactional theory approach discussed herein are of great necessary to guide this study as it has been challenging to comprehend the dynamic interaction process and track the developmental trajectory for young children due to long-lasting concerns about the pandemic crisis. However, researchers are very limited to enter the caring field of ECEC during the COVID-19 pandemic to conduct qualitative research, such as in-depth observations in nature. Thus, it is important to examine the factors influencing teacher-toddler interactions over time in the midst of social controversy about how the quality of teacher-child interaction improves living in a contact-free society as a “new normal.” This may be inferred as seeing a new paradigm in the related research during the socio-historical period of COVID-19.

### Purposes of the study

To fill gaps in the related research discussed so far, the primary research purpose was to examine the potential factors that could enhance the development of toddlers by observing the dynamic interaction patterns between teachers and toddlers during free play time at the center-based ECEC settings in Korea. Furthermore, it focused on how the levels of teachers’ sensitivity affected those patterns of interactions over time. Inspired and guided by the previous study by Bornstein and Tamis-LeMonda (1990), both concurrent and cumulative effects of teachers’ early interaction with toddlers were investigated in terms of stability and change across two time points (Time 1 vs. Time 2, with a four-month interval) in the unique context of COVID-19. This approach will help researchers or practitioners comprehend the new competencies required for young children to reach their fullest potentials living in different daily routines.

### Research hypotheses

To meet the primary purposes of this study, three research hypotheses were developed and tested:

*H1:* Toddlers’ initial experiences (Time 1), including interaction with a teacher and teacher’s sensitivity, could predict the later development (Time 2) and have a long-lasting effect.

Based on the findings that a toddler and teacher’s initial interaction acts as a positive factor that buffers the later developmental risk of toddlers with low SES characteristics (Prime et al., 2020), it is plausible to expect a consistent effect over time on toddler development.

*H2*: Toddlers’ initial experiences (Time 1), including interaction with a teacher and teacher’s sensitivity, could have an immediate effect one toddles’ development simultaneously.

Given that toddlers wear a mask all day in early child education and care settings and interact with peers or teachers through their eyes with their mouths being covered, it would be difficult to comprehend other intentions and induce the stability or change patterns of toddler’s development immediately. Therefore, we expected the main variables’ concurrent impacts on the toddler’s development.

**H3:* Toddlers’ initial (Time 1) and later experience (Time 2), including interaction with a teacher and teacher’s sensitivity at each time, could affect the toddler’s development cumulatively.*

According to life-span theory (Benner and Mistry, 2020), socio-historical events such as the COVID-19 pandemic could drive a toddler’s development depending on life span not only during the same period but also over time in the complex interaction context. Therefore, H3 assumes that a toddler’s development would be affected by related variables at both the initial (Time 1) and later time period (Time 2).

## Methods

### Research design

In order to carry out the research objectives, a non-experimental survey research design was undertaken. Also, the qualitative data was obtained *via* on-site observations by trained researchers. To some extent, this study was semi-longitudinal in nature in that it was designed to examine the impact of socio-historical event of COVID-19 on the interactions between toddlers and teachers over time.

### Participants

The study participants were 63 toddlers aged 2–3 years old and their six teachers in a childcare setting located in Gyeong-gi Province in South Korea. To examine the variables that affected toddlers’ development during the COVID-19 pandemic, this study’s aims and procedures were explained on paper to the mothers, who then signed an informed consent form. At Time 1, the 63 toddlers (37 boys, 26 girls) and their six teachers were observed in their class. The toddlers’ mean age was 40.5 months (*SD* = 8.46). A total of 66.6% of the toddlers were two-years-old (*n* = 42), whereas 33.4% of the toddlers were three-years-old (*n* = 21), respectively. The toddlers’ mean level activity of temperament was 3.94 (*SD* = 0.55), emotionality was 2.76 (*SD* = 0.70), and sociability was 3.57 (*SD* = 0.46). The toddlers’ physical development score was 27.22 (*SD* = 2.20), language/cognitive score was 36.78 (*SD* = 3.90), and sociality score was 33.24 (*SD* = 2.97). Two of the teachers had 2–3 years of college education or less (33.3%), and four of them had a four-year university education or an advanced degree (66.7%). Fifty percent of teachers (*n* = 3) had less than 10 years of teaching experience and 50% of teachers (*n* = 3) had more than 10 years.

After 4 months, 35 toddlers and 4 teachers participated in a dyadic interaction in the same childcare center at Time 2; 28 toddlers were not included because of home-based rearing or difficulty in completing all observation sessions due to the rapid spread of COVID-19. The specific descriptive information on toddler and teacher characteristics is presented in Table 1.

**Table 1:** Descriptive statistics for study subjects.

Variable			*N*	%	*M* (SD)
Toddlers (*n* = 63)	Gender	Male	37	58.7	
		Female	26	41.3	
	Age				40.50 months (8.46)
		2-year-old (28 ~ 38 months)	42	66.6	
		3-year-old (39 ~ 51 months)	21	33.4	
	Temperament	Activity			3.94(0.55)
		Emotionality			2.76(0.70)
		Sociability			3.57(0.46)
	Development	Physical			27.22(2.20)
		Language/Cognitive			36.78(3.90)
		Sociality			33.24(2.97)
		Total			39.48(3.35)
Teachers (*n* = 6)	Education	2–3 years college	2	33.3	
		4-year Univ.	4	66.7	
	Teaching Experience				9.9 months (102.04)
		Less than 10 years	3	50.0	
		More than 10 years	3	50.0	

### Measures

Toddler development. Mothers measured the toddler’s development at home using the K-CDI (Korea version of the Child Development Inventory), which was based on the M-CDI (Minnesota Child Development Inventory: Ireton and Thwing, 1972) and standardized by Kim and Shin (2006) with Korean children. The K-CDI consists of eight dimensions and a total of 270 ~ 300 items: sociability, self-care behavior, gross motor, fine motor, expressive vocabulary, comprehensive vocabulary, letters, numbers. Each item regarding what toddlers could or could not do was rated with three points, with 1 = yes, 2 = no, and 3 = no response. For the present study, these subscales of K-CDI were classified into three dimensions: sociability, physical ability, and language/cognitive ability.

#### Toddler–teacher interaction

To observe the interaction of toddlers with their teachers in a childcare center, the Observational Record of the Caregiving Environment (ORCE) of NICHD Early Child Care Research Network (1996) was used, which had been employed in the study of Seo and Song (2021). The ORCE consists of four motivations (shown in Table 2): physical or physiological, emotional, conflict mediation, and play participation. Two types of toddlers’ interaction behavior were rated: (1) verbal (voice initiation, naming, response, question, request, and in or out of contextual conversation); and (2) behavioral interaction (attention, behavior, imitation, acting on demand, pointing to, nodding, body contact, and laughing/smiles).

**Table 2:** The definition of toddler–teacher interaction.

		Subscales	Content
Motivation		Physical/physiological	Interaction attempted by the teacher in response to the toddler’s instinctive needs
		Emotional	Interaction that toddlers attempt to feel psychological stability from the teacher
		Conflict mediation	Interaction attempted to elicit teacher intervention when toddlers have peer conflicts
		Play participation	Interaction that toddlers try to interact with the teacher for play
Type	Verbal	Voice initiation	Imitate the teacher’s utterance
		Naming	Refer to objects or places such as toys and books
		Response	Respond verbally to the teacher’s questions
		Question	Ask questions to the teacher to induce the teacher’s verbal response
		Request	Asks the teacher for his/her needs or requirements
		In or out of contextual conversation	Talk to the teacher about own thoughts, experiences, situations or related stories
	Behavioral	Attention	Focus attention on the teacher’s words for at least 5 s.
		Behavior imitation	Imitate the teacher’s behavior
		Acting on demand	Act according to what the teacher asked a toddler to say.
		Pointing to	Point to objects or places such as toys or books.
		Nodding at	Nod head in a positive response to the teacher’s words
		Body contact	Make physical contact with the teacher.
		Laughing/smiling	Smile as a positive response to the teacher.

#### Teachers’ sensitivity

The level of teachers’ sensitivity was collected through the same observation tool of the ORCE scale (NICHD Early Child Care Research Network, 1996) which including interactions that teachers initiated to toddlers. We conceptualized interactions that a teacher initiated to toddlers as an aspect of a larger construct of teachers’ sensitivity. The sensitivity of a teacher was organized in four dimensions (shown in Table 3): (a) verbal sensitivity that instructs or provides questions and requests; (b) emotional sensitivity that responds positively, such as expressing emotional/affectionate expressions and praising the toddler’s behavior; (c) developmental stimulus that leads to the upper outcome for cognitive, language, and social development of toddlers; and (d) behavior control that includes restricting toddlers’ behaviors and the physical environment.

**Table 3:** The definitions of sensitivity of teachers.

	Subscales	Content
Verbal	Direct	Give toddlers general routine instructions
	Ask	Ask questions about the toddler’s behavior and related situations
	Request	Ask the toddler for help
Emotional	Express emotions/affections	Express of emotional or affectionate behaviors to toddlers (e.g., “pretty,” “I love you,” petting, hugging)
	Response to the toddler’s behavior (positive or negative)	Express negative verbal expression of toddler’s behavior (e.g., threatening, reacting hostilely, scolding, criticizing, yelling) or positively (speaking affectionately, praising)
Developmental stimulus	Provide developmental stimulation	Support the toddler’s developmental stimuli (cognitive, linguistic, social) by providing instructions, questions, requests and elaborate explanations
Behavioral control	Restrict toddler’s behavior	Restrict behavior by physically separating or moving the toddler (i.e., physical separation, time-out)
	Restrict physical environment	Limit the physical environment around the toddler (i.e., removing toys, no access)
	Show negative/physical behaviors	Negative physical behavior toward the toddler

For the present study, the following data were used in the analyses, except for the teachers’ sensitivity of behavior control and positive response due to the low frequency and negative meaning. Data were collected by trained three observers, who during training had reached agreement at around 85%.

### Procedure

The main variables of this study were assessed through Time 1 and Time 2, respectively. Prior to the main survey at Time 1, a preliminary survey was conducted to explore timely research methods for COVID-19. Regarding the suitability and understanding of the measurement tool, the content validity was verified by one professor of child studies and three experts with childcare experience who are currently in the doctoral program of the graduate school. Also, observers were composed of three graduate doctoral students who received reliability training for six toddlers from 28 to 51 months and two teachers in the childcare center. It was conducted three times until the inter-observer reliability was 85% or higher.

In the first study, S childcare center in Gyeong-gi Province of South Korea was selected as a research institute. The first study was conducted from November 2020 to January 2021 and consent forms for research participation were sent to the mothers, who completed the toddlers’ development and temperament questionnaire. There were 63 toddlers and six teachers who participated to explore the overall characteristics of the toddler’s trial interaction and the teacher’s sensitivity to the toddler’s needs and the development in the free play time situation. Three trained observers observed and recorded the toddlers’ interactive behaviors and teachers’ sensitivities.

However, due to the reoccurring COVID-19 crisis, the observation method was modified to use non-face-to-face observation using a body cam as well as face-to-face observation. Toddler–teacher interaction and teachers’ sensitivity were recorded and analyzed for a total of 160 min in four sessions of 40 min per toddler during morning/afternoon playtime for 63 children aged 28–51 months and six homeroom teachers.

Toddlers’ interactions with teachers were assessed at 40 min per toddler for a total of four times through the free-play time, excluding morning or afternoon lunch, snacks, and outdoor and large group activities. The entire data collection through observation was conducted both through a non–face -to-face method using a body camera and face-to-face by considering social distancing and safety guidelines. All observations were made by three graduate students who had been trained regularly on the measure until achieving at least 85% reliability.

The second study was conducted 4 months after the end of the first study (May to July 2021). Of the 63 toddlers in the primary study, the final 35 toddlers were selected for the secondary study, excluding 28 infants who did not complete the fourth observation period due to family childcare or personal circumstances. The observation method, observation items, and mother-reported questionnaire items were the same as those of the primary study. Moreover, the influence of the major variables predicting toddlers’ development at Time 2 was assessed by examining the stability and change trends of the interaction and teacher sensitivity in the previous period (Time 1) and the post-time period (Time 2).

### Analysis approach

Our primary aims were, first, to describe the study variables (toddler’s development, interaction with teacher, teachers’ sensitivity) and differences according to the characteristics of toddlers (gender, age, and temperament) through a *Mann–Whitney U* or *F* test. In addition, we examined those patterns of change by a *Wilcoxon signed rank test* and stability by a *Spearman rank correlation* across the two time periods (Time 1 and Time 2) for each quality measure. Finally, *stepwise regression* with two-way interaction between toddlers’ interaction and teachers’ sensitivity was performed to identify the potential effect of the study variables on the toddlers’ development over time.

## Results

### Preliminary findings

Descriptive information on toddler’s development, interaction with a teacher, and teacher’s sensitivity is presented in Table 4. As a result of examining the overall interaction motives that toddlers initiated to the teacher, the physical/physiological and play participation motive appeared most frequently while emotional and conflict mediation were observed with low frequency. Therefore, this study preceded with a focus on the two interaction motives (participation in play and physical/physiological motive).

**Table 4:** Descriptive characteristics and tests of differences for study variables.

		Gender			Age			Temperament			
		Boy (*n* = 37)	Girl (*n* = 26)	*t* or *z*	2-year-olds (*n* = 42)	3-year-olds (*n* = 21)	*t* or *z*	Activity (*n* = 14)	Emotionality (*n* = 14)	Sociability (*n* = 35)	*F*
		M(SD)			*M(SD)*			*M(SD)*			
Toddlers’ development	Physical	59.50(8.11)	60.13(6.49)		57.00(6.84)	61.62(7.26)	−0.19	56.57(3.77)	57.90(5.22)	61.15(8.29)	1.25
	Sicio-emotional	65.95(12.99)	64.33(17.91)	0.31	66.64(14.66)	64.33(15.26)	0.44	64.14(14.11)	47.00(23.15)	69.57(10.18)	5.99**
	Language/Cognitive	56.66(8.26)	57.75(8.04)	−0.39	53.00(5.63)	59.88(8.37)	−2.69*	52.68(7.88)	59.75(7.06)	57.91(8.12)	0.24
	Total	53.68(11.91)	52.76(11.60)	−0.37	48.94(10.66)	57.00(11.36)	−2.22*	50.43(8.92)	55.83(13.18)	53.08(12.25)	0.34
Toddler–teacher interaction	Motive										
	Physical/Physiological	1.66(0.92)	1.16(0.77)	−1.72	3.00(3.61)	0.37(0.63)	−0.37	3.50(4.57)	1.55(3.19)	0.76(1.14)	3.32*
	Play participation	12.38(9.91)	11.73(11.63)	−0.43	16.45(11.44)	6.11(2.92)	−2.25*	17.00(11.88)	11.20(9.42)	7.98(7.15)	3.04
	Type										
	Verbal	2.14(1.75)	4.07(4.27)	−0.68	4.91(3.61)	1.55(1.84)	−3.51***	4.39(4.81)	5.35(4.25)	1.92(1.53)	3.13
	Behavioral	3.95(2.42)	5.42(3.39)	−1.20	5.86(3.03)	3.73(2.58)	−2.18*	4.71(3.19)	5.80(3.13)	4.27(2.86)	1.52
Teachers’ sensitivity	Verbal	5.93(4.69)	5.43(6.01)	−0.63	9.93(5.96)	2.86(3.22)	−2.60**	8.36(5.00)	7.90(7.98)	4.39(5.10)	3.92
	Emotional	2.55(2.98)	1.40(1.92)	−1.31	2.32(2.21)	1.24(1.72)	−0.79	2.00(1.55)	2.20(2.71)	1.46(1.97)	1.28
	Development stimulation	3.68(3.31)	2.50(2.71)	−1.01	5.89(5.34)	1.17(1.42)	−2.02*	5.79(4.29)	5.30(7.54)	1.74(2.74)	8.16*
	Total	12.15(9.08)	9.30(9.77)	−1.04	15.07(9.74)	8.17(8.17)	−0.64	14.93(7.82)	12.80(10.37)	9.30(9.49)	2.44

*p < 0.05, **p < 0.01, ***p < 0.001. *n* = 63.

As a result, no differences were found in the main variables across the toddlers’ gender. Three-year-old toddlers showed higher language/cognitive development (*z* = −2.69, *p* < 0.05) and overall development (*z* = −2.22, *p* < 0.05) than two-year-old toddlers. Otherwise, two-year-old toddlers tried to interact more actively with their teachers verbally (*z* = −3.51, *p* < 0.001) and behaviorally (*z* = −2.18, *p*<. 05) than three-year-old toddlers. Also, teachers interacted more verbally (*z* = −2.60, *p* < 0.01) and provided developmental stimulation (*z* = −2.02, *p* < 0.05) with the two-year-old toddlers. Toddlers who had higher sociability of temperament had upper socio-emotional development than toddlers with high emotionality (*F* = 5.99, *p* < 0.01). Finally, toddlers with high activity temperament had more frequent communication by physical and physiological demands than toddlers with emotionality and sociability temperament (*F* = 3.32, *p* < 0.05) and induced higher sensitivity of teachers of providing developmental stimulation to them (*F* = 8.16, *p* < 0.05).

Stability and Changes Time 1 to Time 2.

In order to examine the stability and change over time, descriptive statistics for toddlers’ interaction and teachers’ sensitivity were conducted between Time 1 and Time 2. As shown in Table 5, behavioral interaction (*z* = −2.07, *p* < 0.05) by physical/physiological motivation (*z* = −4.95, *p* < 0.001) at Time 2 exhibited a statistically significant decrease when compared to Time 1. It was also found that a toddler’s verbal interaction with a teacher occurred simultaneously, as observed by both stability (*r_s_* = 0.49, *p* < 0.05) and change (*z* = −3.27, *p* < 0.01). With respect to teachers’ sensitivity, the overall score (*z* = −2.65, *p* < 0.01) and developmental stimulus (*z* = −1.98, *p* < 0.05) indicated a decrease at Time 2.

**Table 5:** Stability and changes on study variables over time.

			Stability (*r_s_*) T1–T2	M (range)		Change (*z*) T1–T2
				Time 1	Time 2	
Toddler–teacher interaction	Motive	Physical/Physiological	−0.18	1.44(0–3.50)	0.23(0–1.88)	−4.95*
		Play participation	0.22	4.36(0–16.38)	4.61(0–14.50)	−0.43
	Type	Verbal	0.49**	5.27(4.74)	2.93(3.15)	−3.27**
		Behavioral	0.18	7.59(8.08)	4.58(2.92)	−2.07*
Teachers’ sensitivity	Verbal		0.02	5.71(5.22)	3.69(2.92)	−1.82
	Emotional		0.05	2.06(2.61)	2.41(2.14)	−0.81
	Development stimulation		0.07	3.17(3.08)	2.06(1.54) 4.38(3.69)	−1.98*
	Total		0.10	13.34(14.28)		−2.65**

*p < 0.05, **p < 0.01. *n* = 35.

### Regression predicting toddlers’ development

To conduct the regression analysis, a Spearman correlation was conducted to examine the predictive relationships between physical and language/cognitive development at Time 2 and relevant variables (toddler–teacher interaction and teachers’ sensitivity), measured at each period. The physical development of the toddler at Time 2 was positively related to the age of the toddler (*r_s_* = 0.45, *p* < 0.01) and sociable temperament (*r_s_* = 0.45, *p* < 0.01) at Time 1. However, emotional temperament (*r_s_* = −0.39, *p* < 0.05), verbal interaction of toddler with a teacher at Time 1 (*r_s_* = −0.38, *p* < 0.05), the behavioral interaction of toddlers (*r_s_* = −0.43, *p* < 0.05), and teachers’ emotional sensitivity (*r_s_* = −0.40, *p* < 0.05) were associated negatively with the toddlers’ physical development at Time 2. In addition, the language/cognitive development at Time 2 had a negative relation to the motivation for play participation (*r_s_* = −0.34, *p* < 0.05), verbal interaction (*r_s_* = −0.37, *p* < 0.05), and teachers’ verbal sensitivity (*r_s_* = −0.43, *p* < 0.05) at Time 1. Indeed, there was a negative relationship with emotional (*r_s_* = −0.48, *p* < 0.01) and developmental stimulation of teacher sensitivity (*r_s_* = −0.41, *p* < 0.05) at the same period. These results highlighted a significant correlation between the toddlers’ development and relevant factors at each time.

Therefore, in this study, three hypotheses were established according to the model based on the method established by Bornstein and Tamis-LeMonda (1990): (1) the effect of toddler–interaction and teachers’ sensitivity at Time 1 on toddlers’ development at Time 2 (Model 1); (2) the effect of the above related variables at the same time (Time 2) on toddler development (Model 2); and (3) the composite effect of toddler–teacher interaction and teachers’ sensitivity in Time 1 and Time 2 on toddlers’ development in Time 2 cumulatively (Model 3).

Only variables that have been reported as statistically related to toddler’s development at T2 physical and language/cognitive development were further investigated. These were entered at each time as independent variables in the multiple regression analyses of the stepwise model selection method to examine predictors of toddlers’ physical and language/cognitive development at Time 2. As indicated in Table 6, significant predictors of toddlers’ physical development at T2 included the following and accounted for 28% of all variances (*F* = 6.15, *p* < 0.01): Toddlers’ behavior interaction with teachers at T2 (*β* = −0.32, *p* < 0.05) and sociable temperament at T1 (*β* = 0.37, *p* < 0.05). In addition, toddlers’ language/cognitive development at T2 was predicted by the following and accounted for 36% of all variables (*F* = 9.10, *p* < 0.01): the toddler’s verbal interaction with a teacher (*β* = −0.44, *p* < 0.01) at T1, emotional sensitivity of teacher at T2 (*β* = −2.20, *p* < 0.05), and the two-way interaction between toddlers’ interaction at T1 and teachers’ emotional sensitivity at T2 (*β* = −0.46, *p* < 0.05). This result indicates that the emotional sensitivity of T2 teachers regulates when the verbal interaction of toddlers to the teacher affects the language/cognitive development of T2.

**Table 6:** Regression analysis for study variables predicting toddlers’ development at T2.

		*R* ^2^	△*R*^2^	*F*	*Β*
Time 2 Physical	(Overall model)	0.28	0.23	6.15**	
	Social temperament T1 (Model 1)				0.37*
	Toddlers’ behavioral interaction with teachers T2 (Model 2)				−0.32*
Time 2 Language /Cognitive	(Overall model)	0.36	0.32	9.10**	
	Toddlers’ verbal interaction with teachers T1 (Model 1)				−0.44**
	Teachers’ emotional sensitivity T2 (Model 2)				−2.20*
	Toddlers’ verbal interaction with teachers T1 × teachers’ emotional sensitivity T2 (Model 3)				−0.46*

*p < 0.05, **p < 0.01. *n* = 35.

Based on these results, it was found that the physical development of Time 2 is explained by “Model 3” (T1 social temperament and T2 toddlers’ behavioral interaction → T2 toddlers’ physical development). In addition, the language/cognitive development of Time 2 was also described as “Model 3″ (T1 toddlers’ verbal interaction, T2 teachers’ emotional sensitivity → T2 toddlers’ language/cognitive development).

**Figure 1:**
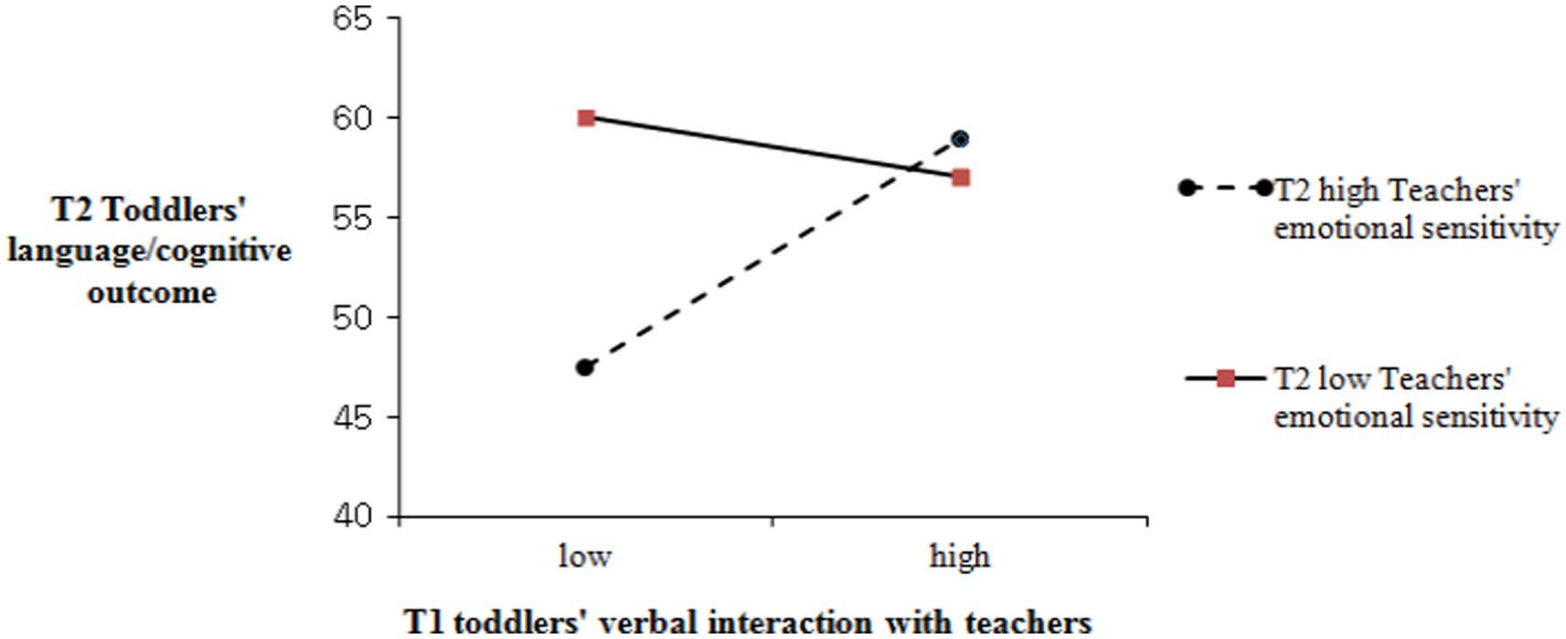
Two-way interaction for T1 toddlers’ verbal interaction with teachers and T2 high teachers’ emotional sensitivity predicting language/cognitive development at T2.

Figure 1 explains the pattern of the two-way interaction by a simple slope test recommended by Aiken and West (1991). It indicates 1 *SD* above and below the sample mean for high and low toddlers’ verbal interaction and teacher’s emotional sensitivity. The test revealed that the association between toddlers’ verbal interaction at T1 and teachers’ language/cognitive development at T2 was significant for toddlers with low teachers’ emotional sensitivity (simple slope: *β* = −0.54, *t* = −2.86, *p* < 0.05) but not for toddlers with high teachers’ emotional sensitivity. These results suggest that the differences in the language/cognitive development in T2 were larger in toddlers who initiated verbal interactions with teachers at T1 according to the emotional sensitivity of the teacher at T2.

## Discussion

The purpose of this study was to examine the potential pathways to toddlers’ physical and language/cognitive development over time that toddlers might experience simultaneously or cumulatively in such a historical event including the current pandemic of COVID-19 based on the life-cycle theory.

As shown in the preliminary results, there were differences in the main variables of interest by child’ age and temperament, but not by his or her gender. First and foremost, a group of three-years old toddlers showed a higher level of language or cognitive development than that of 2 years old ones, as expected. This is in support of the contention from previous studies that as a child grows older, his or her receptive and expressive vocabulary increases as well (Lee and Lee, 2016; Kim and Kim, 2019). Also, temperamentally active toddlers with physical/physiological motives were more likely to be active in order to interact with their teachers, and that could be interpreted to support the argument that infants with a high level of large and small muscle development have strong demands for voluntary behavior and actively move their body, and they tend to express the discomfort they feel by wearing a mask as a positive emotion (Moon and Yi, 2018).

A couple of notes need to be addressed to discuss a distinct pattern between the two groups by a child’ age and temperament in the teacher’s interaction with toddlers. First and foremost, it was observed that a tendency has emerged for teachers to interact in a different and directive way to control toddlers’ behaviors for their health, hygiene and safety due to the changes in the basic routine that toddlers and teachers face after the onset of COVID-19 outbreak (An et al., 2018). Specifically, teachers were more likely to exert immediate verbal controls to the toddlers of 3 years old in order to minimize physical contacts with their peers than the 2 years old toddlers. But, for the toddlers of age of two, teachers were more likely to respond appropriately to their temperamental needs than those of the 3 years old. It could be possible to discuss that distinct finding between the two age groups in that a type of physical activities were strictly restricted in ECEC as a preventive measure of COVID-19 infection and most of play activities were static in nature, such as block-stacking and scissoring a piece of paper, thus improving fine motor skills in the toddlers of this study.

Given that researchers have consistently shed light on the physical or structural dimension related to quality of childcare, the current study confirms the argument that a high ratio of infants to teachers inhibits teachers’ interactions with infants, which is not conducive to positive development in young children (Le et al., 2015; Pessanha et al., 2017). Taken as a whole, it is necessary to examine in-depth the physical elements of childcare facilities in order to improve the overall quality of child care and its impacts on child development.

In terms of the second hypothesis of the current study that toddler–teacher interaction and teacher sensitivity level could be maintained or changed over 4 months in time, the verbal interactions that toddlers had initiated with teachers showed stability even after time passed. In other words, toddlers who had been actively involved in initiating their verbal interactions with teachers during playtime showed more verbal interactions with their teachers even after 4 months passed. Delineated from Sameroff’s exchange theory (Sameroff, 2009), this result is in line with the findings from previous studies that parent–child relationships have dynamic characteristics of exchanging influences over time rather than at a specific point in time. For example, the behavior of 10-month toddlers imitating their mother’s voice increased over time (Markodimitraki and Kalpidou, 2021), and the level of vocabulary in 18-month toddlers predicted that of 24 months of age in toddlers later (Suttora and Salerni, 2011). Expanding this result obtained from the family context to that of daily routines in ECEC, the patterns of interaction between toddlers and teachers reflect the mechanism that shares social and emotional shared meanings and integrates dynamic elements in nature.

Meanwhile, the finding pertaining to the decreased change in physical, physiological, verbal, and behavioral interactions among toddlers after a 4 months lapse supports the research by Ha and Seo (2011) who found that positive interactions, such as asking questions to teachers or imitating teachers’ behaviors from 12 to 30 months, decreased rapidly over time. In accordance teachers also showed a decrease in their levels of sensitivity that may induce developmental stimulation for toddlers at Time 2, as compared to those observed earlier at Time1. There are inconsistent findings over the teachers’ sensitivity as a child grow older. On the one hand, teachers could more easily grasp toddlers’ signals for communication and meet their needs by responding verbally as toddlers improve their levels of language, cognition, and physical development over time (Deynoot-Schaub and Riksen-Walraven, 2008). On the other hand, it is expected that older toddlers are more likely to interact with their peers than teachers from a perspective of peer scaffolding during free play (Shin, 2009; Pessanha et al., 2017). Thus, the interaction patterns vary by contexts of subject, time and history, it would be necessary to understand the multi-faceted impacts of the pandemic on toddler development.

In this study the third hypothesis was tested to investigate how the toddlers who have experienced a certain social and historical event of COVID-19 interact with the nested environments and that, in turn, affects both the concurrent and predictive outcomes in toddlers. To process this, the current study employed the three developmental paths, suggested by Bornsteem and Tamis-lemonda (1990) from the perspective of Life Cycle Theory (Elder and Shanahan, 2007), which assumes that the everyday context experienced by the interaction subject can be internalized and cause a turning point in the development trajectory. The focused areas to track the development path in this study were only the physical and language/cognitive development of toddlers. As rapid brain development occurs in the early stages of life, the speed of information transfer between cells increases, and the coordination of eyes and hands and motor skills improves to acquire appropriate social skills for each development trajectory and expand interaction targets, thus it is assumed that toddler physical and language/cognitive development can be clearly observed, as compared to other areas of social and emotional development, which is a psychological factor of toddlers and is relatively more stable after a short time lapse (Benner and Mistry, 2020).

Based on the three paths presented by Bornstein and Tamis-lemonda (1990), the paths of major variables affecting the physical development of toddlers in subsequent periods (T2) over 4 months were explored. As a result, it was found that the early (T1) social disposition of toddlers and the behavioral interaction that toddlers had initiated with teachers revealed a significant effect, supporting both models 1, 2, and 3 respectively, which are simultaneous, cumulative, and complex paths, and discussed in detail as follows. On the one hand, toddlers with high social temperament characteristics in the early stages of their lives are likely to actively attempt nonverbal interactions, such as reaching out to catch interesting playgrounds or attempting collaboration with teachers (e.g., staring). This is consistent with the results of several recent previous studies (Longobardi et al., 2014; Cho, 2018) that revealed that as physical autonomy increases, more people and objects are observed and participated in physical activities. Furthermore, it can be inferred that toddlers with high social temperament had abundant opportunities to gradually improve physical development, such as sitting and walking, to try to interact with others in a way appropriate to their developmental characteristics over time and to utilize social context clues.

On the other hand, the level of physical development of toddlers was predicted to be higher for toddlers who attempted less behavioral interaction with teachers at the same time (T2). This is inconsistent with the findings of Liszkowski and Tomasello (2011), who reported that the interaction intention becomes clear in the late 1st year of age and has a positive effect on toddler development by attempting to interact in the form of nonverbal movements such as pointing to objects. However, in the late second year of the study, as toddler autonomy increases, peer participation increases more than teacher interaction, and in this process, we support the research results of Recchia and Shin (2012) and Cho (2018), which reported the characteristic of gradually transferring nonverbal movements to rich verbal expressions.

Also, it is noteworthy to address the paradigm shift in ECEC centers implemented during the pandemic period to support the results related to the negative effects of behavioral interactions in toddlers on simultaneous physical development. To prevent COVID-19 infection, as large groups and outdoor activities were entirely banned in ECEC, and static play activities in the form of small groups were recommended, toddlers frequently interacted with peers or teachers in the form of small groups in the classroom. Therefore, it can be assumed that the delicate play that requires small muscle exercise skills, such as stacking blocks, engaging in role plays, or using scissors, which are centered on small groups has also affected the physical development of toddlers.

Next, it was found that the verbal interaction of early (T1) toddlers and the sensitivity of contemporary (T2) teachers negatively affect the language/cognitive development of contemporary toddlers, respectively, which can be seen as supporting all three hypotheses assuming continuous, simultaneous, and cumulative influence. Specifically, in the case of toddlers who try less verbal interaction with teachers in the early stages of observation, the result of higher language/cognitive development after 4 months is the result of supporting Hypothesis 1, which suggests continuous influence, and can be examined in the same context as a study by Cho (2018), who revealed changes in the pattern of interaction motivation by teachers as a child’s age increases. In other words, in the early stages of toddlers, toddlers try to interact with their teachers to meet needs for cognitive and emotional motivation by asking for help to express their emotions, but the patterns for toddlers’ interaction motivation to interact with teachers change in order to facilitate their levels of engagement in play with peers. Therefore, toddlers who try less verbal interaction with teachers in the early stages of observation show a higher level of language development, thus enabling them to communicate verbally with peers at their early age.

Furthermore, the low emotional sensitivity of teachers at the same time supports Hypothesis 2, which emphasizes immediate effects by predicting the language/cognitive development of toddlers at a higher level. Until now, the importance of responsive sensitivity of teachers has centered on the issue of quality of ECEC, and been universally agreed upon in the academic arena (Seo et al., 2016). Unlike previous studies, which consistently have found that the emotional sensitivity of teachers experienced by toddlers had a positive effect on acceptance, expression, self-regulation, and cognitive development at the age of two (Cadima et al., 2010; Kim and Kim, 2019; Davies et al., 2021), the current study revealed teachers in ECEC settings focused their interactions with children to provide tailored services to coordinate toddlers’ words and actions, mainly on issues related to safety, healthy, and hygiene (An et al., 2018). In that process, toddlers with a high level of language/cognitive development were more able to initiate verbal interactions with their peers in order to expand the scope of social relationship formation, while toddlers with lower levels of language development experience with difficulty to express a variety of emotions in relationships with peers (Han, 2021). Therefore, in order to motivate toddlers to participate in play with their peers, teachers ought to be required to create a positive atmosphere where teachers could react sensitively to toddlers in needs or express affection at individual levels.

In a support of the Model 3, the linguistic interactions initiated by toddlers with teachers in T1 and the emotional sensitivity of teachers in T2 showed interaction effects, predicting the language/cognitive development of toddlers at the same time (T2). Given that the emotional sensitivity of teachers at T2 would play a moderating role in the relation between the linguistic interactions of toddlers at T1 and the language/cognitive development at Time 2, the claim for interpreting this fining needs to be provided with caution in that teachers did not react sensitively to or tune-in to the emotional needs of toddlers in the process of attempting verbal interactions with toddlers under the COVID-19 circumstances. Interestingly, if the emotional sensitivity of the teacher at Time 2 and the frequency of verbal interaction attempted by toddlers at Time 1 are low, then it will have the most positive effect on the language/cognitive development of the toddler at Time 2. Under the COVID-19 circumstances, toddlers and teachers ought to wear their masks all day long, and it was limited to interact verbally with toddlers, teachers found an option or alternative to catch non-verbal clues including gestures, facial expressions, and body signs from toddlers in order to meet the needs of toddlers in more adaptive ways.

As expected, for the toddlers who had frequent interactions with teachers (both verbally and non-verbally) in the early period (T1), their levels of language or cognitive development were found to be high at the later period (T2), regardless of the levels of teachers’ emotional sensitivity at the same time (T2). In the social context where toddlers belong to, they experience a variety of communication opportunities by imitating the teacher’s verbal expression (Jung and Kim, 2015). Also, it is in line with the research by Kirk et al. (2013) who found that the close interactions initiated by toddlers with teachers at the very early age, which were coordinated or tuned-in by sensitive teachers to meet the needs of toddlers had a positive effect on their vocabulary development at a later period. Thus, this study supports the premise from the transaction theory and during the COVID-19 period in general, and the teachers’ sensitivity not only to the diverse needs of young children, but also to mandatory regulations in the historical and social contexts.

In sum, the above discussion suggests that sensitive interactions with teachers experienced early in life can have a major impact on the development of post-toddler periods (Blewitt et al., 2020), and that teachers’ emotional support appropriate for individual toddlers’ development levels and characteristics can serve as a driving force for toddler development. Therefore, the socio-historical change of COVID-19 suggests that in order to provide developmental support to toddlers as a required competency for teachers in the childcare field, they should recognize the importance of early and cumulative experiences and respond sensitively for toddlers to grow as active communicators according to the development trajectory.

## Data availability statement

The original contributions presented in the study are included in the article/supplementary material, further inquiries can be directed to the corresponding author/s.

## Ethics statement

Ethical review and approval was not required for the study on human participants in accordance with the local legislation and institutional requirements. Written informed consent to participate in this study was provided by the participants’ legal guardian/next of kin.

## Author contributions

SJS was contributed as both the primary investigator and corresponding author for this research. JYS was contributed as co-investigator to assist collecting and analyzing the data for this research. All authors contributed to the article and approved the submitted version.

## Conflict of interest

The authors declare that the research was conducted in the absence of any commercial or financial relationships that could be construed as a potential conflict of interest.

## Publisher’s note

All claims expressed in this article are solely those of the authors and do not necessarily represent those of their affiliated organizations, or those of the publisher, the editors and the reviewers. Any product that may be evaluated in this article, or claim that may be made by its manufacturer, is not guaranteed or endorsed by the publisher.
